# Supplementary material for: Multiplex short tandem repeat profiling of immortalized hepatic stellate cell line Col-GFP HSC
Source: PLoS One. 2022 Sep 6;17(9):e0274219. doi: 10.1371/journal.pone.0274219 (PMC9447916; doi:10.1371/journal.pone.0274219)
Supplement: Attachment — Submitted filename: review_comments.docx [file pone.0274219.s001.docx]

The research article entitled ‘Multiplex Short Tandem Repeat Profiling of Immortalized Hepatic Stellate Cell line Col-GFP HSC’ by Steffen K. Meurer et al provides STR profile for identification of mouse cell line termed as-Hepatic Stellate Cell line Col-GFP HSC. This cell line is an important tool to investigate fibrogenic signalling especially using PDGF, TGF-β1 etc. The reported information in this paper is useful to wider researcher community working with the different mouse/human cell lines. Therefore, I recommend the paper for publication, however following issues need to be addressed.

1. It is difficult to read the figure1 therefore use better resolution of figure 1.
2. It may be advised to rephrase the sentence ‘In this study… cell line authentication (line 15-16)’. It gives impression that this sentence is refering to PLoS One paper in the year 2013.
3. Line 58 check spelling of ‘stimuli’.
4. Table 1- What does the ‘allele range mean’? It may be mentioned in the table legends or may be inserted in the text somewhere.
5. Table 1- if the information on the primers and PCR size is available/ or in the public domain then it may be inserted.
6. Insert some statement on the significance of STR profile homologies. Refer line 123-127.
7. Provide the information on what the different numbers in the table 2 refer to? and what they signify?
