# Supplementary material for: Multiplex short tandem repeat profiling of immortalized hepatic stellate cell line Col-GFP HSC
Source: PLoS One. 2022 Sep 6;17(9):e0274219. doi: 10.1371/journal.pone.0274219 (PMC9447916; doi:10.1371/journal.pone.0274219)
Supplement: Attachment — Submitted filename: Rebutal letter.docx [file pone.0274219.s002.docx]

**Reviewer #1**

The research article entitled ‘Multiplex Short Tandem Repeat Profiling of Immortalized Hepatic Stellate Cell line Col-GFP HSC’ by Steffen K. Meurer et al provides STR profile for identification of mouse cell line termed as-Hepatic Stellate Cell line Col-GFP HSC. This cell line is an important tool to investigate fibrogenic signalling especially using PDGF, TGF-β1 etc. The reported information in this paper is useful to wider researcher community working with the different mouse/human cell lines. Therefore, I recommend the paper for publication, however following issues need to be addressed.

*Dear Dr. Nitharwal,*

*Many thanks for the thoughtful review of our work and your critique. We have addressed your comments as stated in the point-to-point response. In the revised version we have marked all changes in red letters.*

1. It is difficult to read the figure1 therefore use better resolution of figure 1.

*We fully agree with your concern. In the pdf file that you reviewed the overall quality of figure 1 was insufficient. As suggested, we have increased the resolution of respective figure and hope that it now looks better in the pdf file that is manually produced by the online submission system. However, please note that the resolution in the pdf file generated by the online system is set to low resolution. For final quality refer to the TIF file of the image.*

2. It may be advised to rephrase the sentence ‘In this study… cell line authentication (line 15-16)’. It gives impression that this sentence is referring to PLoS One paper in the year 2013.

*Thanks for this suggestion. We have rephrased the mentioned sentence to “In the present study …..”.*

3. Line 58 check spelling of ‘stimuli’.

*We are sorry for this spelling error and corrected the term in the revised version.*

4. Table 1- What does the ‘allele range mean’? It may be mentioned in the table legends or may be inserted in the text somewhere.

*That is a great suggestion. We have now explained the term “allele range” in the footnote of table 1 (now table 2).*

5. Table 1- if the information on the primers and PCR size is available/ or in the public domain then it may be inserted.

*We have now compiled a new table in which we have added the primer information. In the new Table 1 we have added a footnote about the sources of primer sequences. All other tables were lined up.*

6. Insert some statement on the significance of STR profile homologies. Refer line 123-127.

*This is an important comment. For computation of the significance of a STR profile, we used the Tanabe score, which is also known as the Sørensen-Dice coefficient (see Capes-Davis et al., 2012). This score is used to gauge the similarity of two samples and is defined as follows:*

*Percent match = (number shared alleles x 2) / (total number of alleles in the questioned profile + total number of alleles in the reference profile)*

*The similarity can range between 0 (completely different) and 1 (identical).*

*The highest homology of Col-GFP HSC was found to cell line AT-3 (score= 0.8293).*

*To clarify this issue we have added a short sentence explaining the Tanabe score in the Material and method section and added the respective reference (new reference 10).*

7. Provide the information on what the different numbers in the table 2 refer to? and what they signify?

*The different numbers refer to the allele constellation at respective loci in the mentioned cell lines. We have added a short comment on that issue in the footnote of respective table (now table 3).*

**Reviewer #2**

This is a nice efforts by authors to authenticate their own cell line. However, there are serious lapses in introduction and discussion part.

*Dear Dr. Singh,*

*Many thanks for the thoughtful review of our work and your critique. We have addressed your comments as stated in the point-to-point response. In the revised version we have marked all changes in red letters.*

1. Authors may discuss pros and cons of various cell line characterization methods, perhaps a tabular comparison will be nice.

*We not attempt to provide a review about cell line characterization methods. However, to address your comment, we have added some brief comments at the end of the discussion section. In addition, we have added a reference in which pros and cons of various cell line characterization methods are discussed.*

2. there is no karyotyping or any other supporting data regarding the cell line. How does the karyotypes look in low passage versus high passage cells, specially focusing on any major abnormalities. it would be very nice if authors could include 2 karyotypes.

*We have recently published two other papers in which we provide karyotypes for the murine cell line GRX (Schröder et al., Cells 2022;11(9):1504) and the rat cell line HSC-T6 (Nanda et al., Cells 2022;11(11):1783). We have not made any attempt to provide a karyotype analysis in this paper because we have now deposited this cell line in a nonprofit cell repository (DSMZ,* [*https://www.dsmz.de/*](https://www.dsmz.de/)*). They will now further characterize this cell line before sending them out to laboratories who request this cell line. However, they requested from us to provide a STR profile that can be used as a reference for this cell lines. This was done in our paper without providing information about the karyotype. However, to address your concern we have added a short comment on the necessity of a karyotype analysis at the end of the discussion section.*

3. There is no discussion about the results. please elaborate. Also readers would like to know if you have/planned to develop any PCR multiplex for the characterization of your cell line.

*The assay that was used for genotyping is a PCR multiplex assay. We have added the respective primers used in this assay in new table 1. All other tables were lined up.*

**Reviewer #3**

Major comments

The authors have profiled the cell line using STR markers which will help to identify the cell line in future. However, this information alone is not sufficient enough to be published as an independent article. This needs to be clubbed with some additional data such as comparison between cell lines of same origin or other related cell lines used by researchers and the identification of any unique marker(s) that will help to differentiate these cell line from one another.

*Many thanks for the thoughtful review of our work and your critique. It is a pity that you are not as enthusiastic as the other two reviewers. We hope that we can convince you with the following presentations that our paper is worth to be published.*

*As you might know, the animal welfare law has dramatically changed in the EU. Therefore, there is an urgent need to have well-characterized cell lines for biomedical research. This is the reason why we recently established the genetic characteristics of murine hepatic stellate cell line GRX (Schröder et al., Cells 2022;11(9):1504) and rat hepatic stellate line HSC-T6 (Nanda et al., Cells 2022;11(11):1783). As we discuss in our short report, the cell line Col-GFP HSC was established in our lab several years ago. After all these years, we now receive many requests for this cell line. In particular, several scientists will use this cell line in cell tracking experiments, which is very simple with our cells because they carry a GFP reporter cassette.*

*The STR profile established in the present study is unique and allows rapid authentication of this cell line. We want to prevent that somebody use unauthenticated cells from our laboratory. As you might know the actual Register of Misidentified Cell Lines currently lists already 576 cell lines that are misidentified (*[*https://iclac.org/databases/cross-contaminations/*](https://iclac.org/databases/cross-contaminations/)*). It is well accepted that misidentified or contaminated cell lines will waste valuable time and resources on experiments and provide the basis for flawed publication. With the STR profile that we have established everybody working with this cell line will be now able to provide the required cell authentication that is requested by many journals and funding agencies. This is the intention of our short communication.*

*We hope you can follow us in our argumentation.*

Minor comments: Repetition of text at several places.

*We have tried to remove unnecessary redundancies.*
