# Supplementary material for: Multiplex short tandem repeat profiling of immortalized hepatic stellate cell line Col-GFP HSC
Source: PLoS One. 2022 Sep 6;17(9):e0274219. doi: 10.1371/journal.pone.0274219 (PMC9447916; doi:10.1371/journal.pone.0274219)
Supplement: Attachment — Submitted filename: Response to reviewers.docx [file pone.0274219.s003.docx]

**Reviewer #1:**

The revised version of the manuscript 'Multiplex Short Tandem Repeat Profiling of Immortalised Hepatic Stellate Cell line Col-GFP HSC' addresses all the specific concerns/points that were raised. The manuscript is recommended for publication.

*Thanks again for all your input.*

**Reviewer #3:**

It was quite unprofessional to use such a language. There is no need to take pity on anyone. My comments were professional and I stand by my comments. I have not questioned the quality of this study but didn't find the study enough to be published as an independent article. If others have recommend your article for publication as such it is completely their choice.

Dear reviewer #3,

*We fully understand your displeasure. However, we had not the intension of taking pity on anyone. Quite the opposite, we are really grateful that you and the other reviewers helped to improve the quality of our work. We respect your opinion that you think that our study does not provide significant data to allow its publication. We agree that we only show a STR profile for the cell line that we established several years ago. In this regard our study is a small “add-on” to the previous paper that was published already nearly a decade ago (Meurer et al., PLoS ONE 2013;8(2):e56116). I guess everybody will realize that our paper is not a classical full paper but just a kind of addition. Nevertheless, this addition is very important because we have now deposited this cell line in a nonprofit cell repository (DSMZ,* [*https://www.dsmz.de/*](https://www.dsmz.de/)*). This will allow every researcher to obtain this cell line for research purposes. However, it is now well-accepted that a defined STR profile that allows easy cell authentication is mandatory when working with a cell line. This is the reason why we would like to publish our addition in the journal in which we originally described the generation of this continuous growing cell line.*

*We hope you understand our argumentation and are sorry if you think that we wanted to tackle you.*

**Reviewer #4:**

Steffen Meurer and colleagues improved their manuscript entitled “Multiplex Short Tandem Repeat Profiling of Immortalized Hepatic Stellate Cell line Col-GFP HSC” (PONE-D-22-11946R1) and addressed major points raised by the reviewers. There are only a few minor aspects that the authors should address to further improve their manuscript:

*Dear reviewer #4,*

*Many thanks for the time you spent in reading our short article. We are grateful for your thoughtful comments. In the revised version, we have addressed your minor comments and marked all changes in red letters.*

General remark: An own analysis of the STR by the authors would have been better for this paper, because then a detailed protocol for the STR analysis would be available for the readers in the section "Material and Methods".

*Yes we initially thought about doing that. However, we then realized that the customized 19 STR markers enclosed in the CellCheck Mouse 19 panel that were used in our study are essentially those that were proposed by the Consortium for Mouse Cell Line Authentication to validate STR markers for intraspecies identification of mouse cell lines (Almeida et al., PLoS ONE 2019;14(6):e0218412). This panel is nowadays widely applied in many laboratories and provides information on all markers that are necessary to allow a quick comparison with STR profiles form other cell lines at the Cellosaurus STR database (*[*https://web.expasy.org/cellosaurus-str-search/*](https://web.expasy.org/cellosaurus-str-search/)*). In our view, offering a protocol of our own would be rather confusion that purposeful.*

A) Line 64: “for the cell line” (was used twice in one sentence)

*We have rephrased the mentioned sentence in line 64.*

B) Line 78: A 100 mm² culture dish does not exist and is certainly too small for 8 million cells. Probably a 10 cm (diameter) is meant here(?).

*You are correct. It must read “10 cm dish”. We have corrected this error.*

C) Line 81: “of Col-GFP HSC cells” (without cells). The abbreviation already contains the word cells.

*Thanks for this comments. We have deleted “without cells”*

D) Line 89: The term “PIGtail” must be defined and explained. Is it a 7-nucleotide tag?

*Yes, in the protocol it refers to a 7-nucleotide tag with the sequence “GTTTCTT”. According to your advice, we have added this information.*

E) Line 113: The term SV40 was already mentioned in the section “Introduction” (line 58). The abbreviation in the line 113 now differs from the former. A detailed description of SV40T should be given when mentioned first.

*Many thanks for this comment. In the revised version, we have now introduced the abbreviation SV40T upon first mentioning and harmonized the abbreviation to SV40T for Simian virus large T antigen.*

F) Line 154: Analysis of “interspecies contamination” was mentioned several times in the manuscript but was not shown by the authors. At least one appropriate citation should be given by the authors at this point.

*Yes this is correct. We have added a new reference (Masters et al., Proc Natl Acad Sci U S A 2001;98:8012-7) and again cited the two references of Ameida et al. (Refs 6 and 7) that discuss that STR profiling is suitable to detect interspecies contamination in human and mouse cell lines.*
